# Anti‐Biofouling Coatings Based on Ultra‐Slippery Surfaces

**DOI:** 10.1002/cbin.70065

**Published:** 2025-07-31

**Authors:** Alexander B. Tesler, Wolfgang H. Goldmann

**Affiliations:** ^1^ Department of Biophysics Friedrich‐Alexander‐Universität Erlangen‐Nürnberg Erlangen Germany; ^2^ Department of Materials Engineering, Faculty of Engineering Bar‐Ilan University Ramat‐Gan Israel

**Keywords:** aerophilic surfaces, air plastron, anti‐biofouling coatings, lubricant‐infused slippery surfaces, nontoxic

## Abstract

Traditional treatment of biofouling with toxic paints or antibiotics has significant limitations and challenges, including negative impacts on surrounding ecosystems and the emergence of resistant microbial strains. Antibiotics often prove ineffective in penetrating the dense and protective structure of biofilms, rendering traditional antimicrobial approaches less effective and leading to chronic infections. Toxic paints, while initially effective in reducing microbial colonization, contribute to long‐term environmental contamination and harm non‐target organisms. In contrast, novel technologies such as aerophilic surfaces, a special type of superhydrophobic surface, and liquid‐infused slippery surfaces offer promising alternatives to conventional biofilm management technologies. While aerophilic surfaces create a physical barrier that inhibits biofilm formation by reducing the direct contact of aqueous media with solid surfaces, liquid‐infused slippery surfaces enhance the anti‐biofouling effect by maintaining a protective lubricating layer that prevents organisms from settling. These nontoxic technologies not only provide a more sustainable and effective means of combating biofilms but also minimize the environmental impact associated with conventional treatments. By leveraging the unique properties of advanced materials, we can increase the durability and effectiveness of surfaces, leading to improved outcomes in various fields, including medical devices and marine applications.

**Abbreviations**
AF: anti‐biofouling
APhS: aerophilic surfaces
FR: fouling release
LISS: lubricant‐infused slippery surfaces
PDMS: polydimethylsiloxane
Plastron: air trapped in metallic protrusion when immersed in water
SHS: superhydrophobic surfaces
SLIPS: slippery liquid‐infused porous surfaces
Ti: titanium

Biofouling is a global problem affecting nearly all water‐based processes and industries (Callow 1993; Schultz et al. 2011). Marine shipping accounts for over 90% of global trade and is responsible for approximately 3% of greenhouse gas emissions (Wu et al. 2022). Biofouling of marine organisms on ship hulls, meanwhile, increases CO_2_/SO_2_ emissions by over 40% due to increased friction (Pulletikurthi et al. 2023). In healthcare, biofouling causes infections in medical devices, resulting in reoperation rates of up to 30% and costing billions of dollars per year in the U.S. (Blomstrom‐Lundqvist and Ostrowska 2021; Evans 2009). Also, bacterial fouling contributes to antimicrobial resistance, posing a major threat to human health worldwide and leading to millions of deaths every year (Cámara et al. 2022; Murray et al. 2022; Poudel et al. 2023).

Biofouling, and marine fouling in particular, is a multi‐step process initiated by the formation of a “conditioning layer” through the adsorption of organic molecules, followed by the attachment of microfoulers, including bacteria, diatoms, and microalgae (Védie et al. 2021). The attached cells promote the formation of the biofilm matrix on which multicellular micro‐ and macrofoulers can thrive (Qian et al. 2022). Early marine antifouling coatings (mostly paints) were developed to prevent colonization by marine organisms and contained toxic compounds such as arsenic or tin that acted as broad‐spectrum biocides (Dafforn et al. 2011; Lejars et al. 2012). However, the environmental impact of these compounds on aquatic life, especially targeting non‐specific surrounding organisms, became apparent, and they were banned in 2008 (Kirschner and Brennan 2012).

Currently, antifouling coatings comprise two main strategies: (i) Anti‐biofouling (AF), which uses chemically active biocidal coatings containing Cu or Zn, organic complexes, and enzymes that act on marine organisms (Anisimov et al. 2019). However, the rate of release of soluble species from these coatings is now subject to regulation in several countries, including the US and EU (Martins et al. 2020). (ii) Nontoxic fouling release (FR) coatings, which either inhibit the settlement of fouling organisms or enhance the release of settled organisms without involving chemical reactions or toxic chemicals (Lejars et al. 2012). The nontoxic FR strategy is inspired by natural organisms such as shark skin (Schumacher et al. 2007) or mollusk shells (Bers and Wahl 2004) in their defense against biofouling and involves the formation of a rough microscale topography on the surface. The FR strategy is based on minimizing the adhesion between fouling organisms and the surface, often by introducing an additional gas‐liquid or liquid‐liquid interface that increases the slipperiness of the surface so that fouling can be removed by simple mechanical cleaning, such as a water jet, or by hydrodynamic stress during ship navigation. The FR surfaces offer extended antifouling life, but commercially available materials based on silicon or fluoropolymer binders limit their application to certain working conditions and make them susceptible to mechanical damage and costly (Lejars et al. 2012; Liu et al. 2023; Zang et al. 2024).

Several FR approaches have been developed in the last decades, mainly mimicking natural surfaces. One such approach imitates the leaves of *Nelumbo*, the lotus flower, which has a hierarchical surface roughness consisting of microscopic papillae overlaid by nanostructured wax epicuticular tubules (Neinhuis 1997). The combination of high roughness and a hydrophobic wax coating causes water to contact the tops of the textured structures, resting mostly on the air trapped within the micro‐nanostructures. This is known as the Cassie‐Baxter wetting state, while the layer of trapped air within rough protrusions is known as plastron (Cassie and Baxter 1944). Such air‐trapping surfaces are commonly referred to as superhydrophobic surfaces (SHS) (Marmur and Kojevnikova 2020). For many years, SHS have been considered as a potential solution for anti‐biofouling surfaces (Genzer and Efimenko 2006). However, the “*Achilles heel*” of SHS is (*i*) the poor mechanical and compressive stability of the hierarchical micro/nano‐scale surface topography, which is essential for achieving very high water contact angles, and (*ii*) the metastability of the trapped air layer underwater, which makes these surfaces short‐lived for biofouling applications (Hwang et al. 2018).

Recently, non‐toxic anti‐biofouling coatings based on aerophilic surfaces, a special type of SHS with a stable plastron underwater, have been demonstrated (Tesler et al. 2023). The approach combines high surface roughness with a hydrophobic coating. The resulting surfaces can trap the air layer within rough protrusions, leading to the development of an additional gas‐liquid interface that covers > 99% of the surface (Figure 1a). This, in turn, prevents adhesion of the conditioning layer, the first step in the biofilm formation process, and misleads biological species to recognize the underlying surface as a solid material. Medical grade Ti‐alloy sheets and rods have been electrochemically patterned with high uniformity over a large area and coated by self‐assembly using a fluorinated phosphate ester surfactant that forms covalent bonds with the substrate. A novel experimental framework has been developed and applied to characterize aerophilic surfaces and demonstrate their thermodynamic stability in underwater environments (Tesler et al. 2023; Tesler et al. 2024). For more details about the thermodynamic stability frameworks, the reader is referred to the following literature (Lafuma and Quéré 2003; Marmur 2006). However, there are still many challenges in characterizing such high area surfaces due to their extreme slipperiness, which renders conventional goniometric measurements unreliable (Prado et al. 2024).

**Figure 1:**
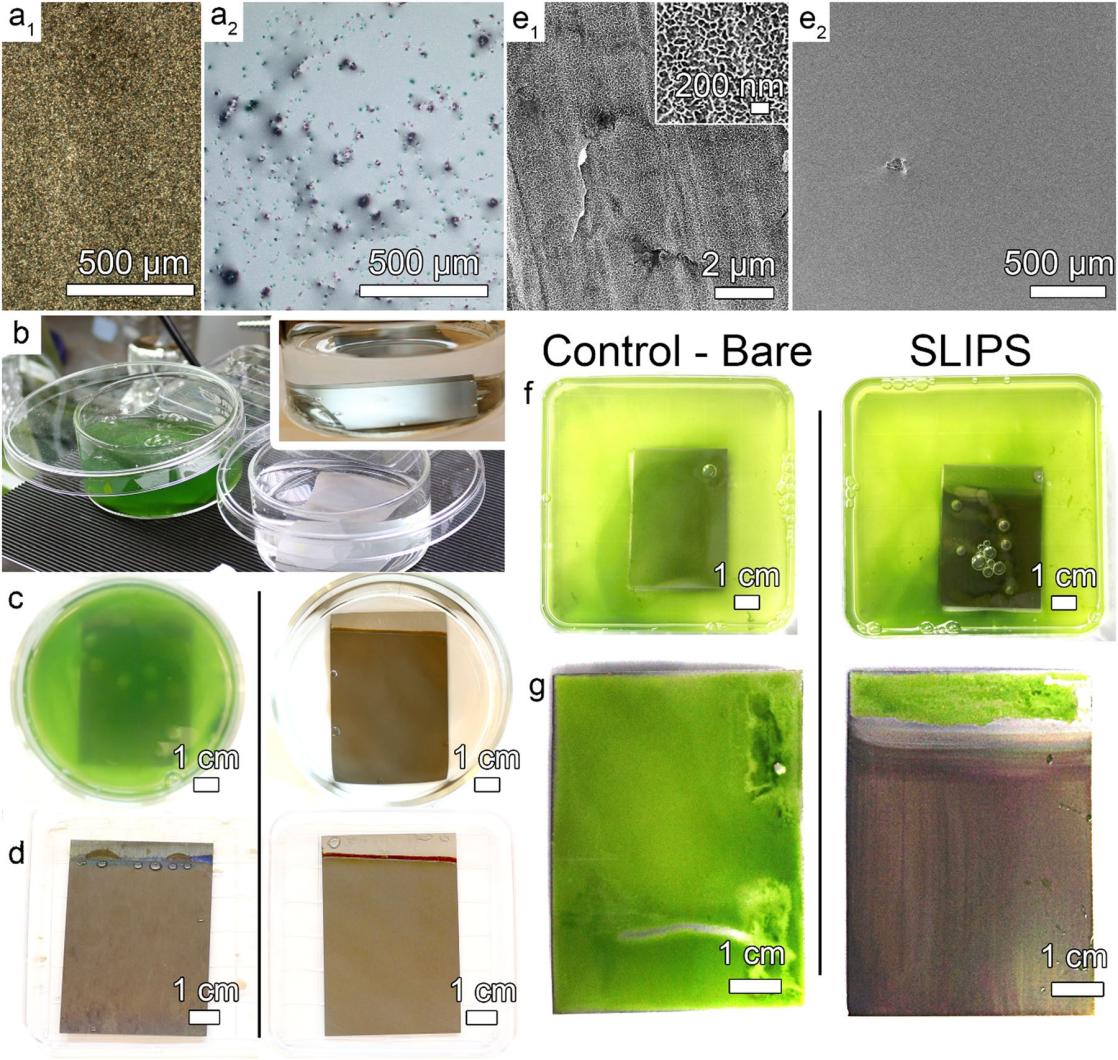
(a) Bright‐field optical reflectance microscopy images of aerophilic Ti alloy surfaces as they appear in air (a_1_) and underwater (a_2_). The bright gray flat areas in (a_2_) represent the water‐air interface, while dark round spots are attributed to the solid‐liquid area, that is, actual contact between water and Ti alloy surface. (b) An anti‐biofouling experiment with aerophilic samples immersed in either deionized water or *Chlamydomonas reinhardtii* (*C. reinhardtii*) growth medium for 10 days. The inset image was captured at the grazing angle to demonstrate the high reflectivity of the Ti alloy sample after 10 days of immersion underwater due to the existence of plastron, that is, the phenomenon occurs due to the total internal reflection of light within a thin air layer. Digital still images of the Ti alloy samples just before (c) and after (d) the harvesting. The harvesting was performed by passing the samples through a water‐air interface and drying them in an ambient atmosphere. Comparably clean surfaces were obtained in both cases. For more details on the formation of aerophilic Ti alloy surfaces, the reader is referred to (Tesler et al. 2023). (e) Scanning electron microscopy images of the tungsten oxide monohydrate rough coating, electrodeposited on AISI316 austenitic stainless steel (e_1_), was coated with a self‐assembly monolayer of fluorinated surfactant and infused with fluorinated lubricant to form SLIPS (e_2_). (f) Still digital images of bare (left, control) and SLIPS (right) samples were immersed in *C. reinhardtii* growth medium for 8 days, then were harvested similarly to aerophilic surfaces by passing them through a water‐air interface and dried in ambient atmosphere. (g) While bare samples were completely covered by *C. reinhardtii* biofilm (left image), the SLIPS samples demonstrated spontaneous delamination of the algae film that was only weakly attached to the surface, leaving behind a fouling‐free surface (right image, brown area). For more details regarding the preparation of the tungsten oxide monohydrate steel samples, the reader is referred to (Tesler et al. 2015).

In a groundbreaking study, we applied a theory to achieve long‐term thermodynamic stability of aerophilic surfaces underwater and demonstrated for the first time that such surfaces can delay the accumulation of bacterial biofilms, completely prevent the attachment of complex fluids such as blood, and marine microfouling organisms such as green algae (Figure 1b–d), as well as macrofouling organisms including mussels, and significantly reduce the attachment strength of barnacles (Tesler et al. 2023). The approach of creating highly rough, hydrophobic solid surfaces with well‐defined uniform morphology and continuous, long‐lasting plastron will serve us in the future as an excellent scientific model to study the protective performance of aerophilic surfaces over months to years for highly demanding anti‐biofouling and anticorrosion applications.

Given the metastability that characterizes the vast majority of SHS and the resulting brevity of their anti‐biofouling protection period (Hwang et al. 2018), the slippery liquid‐infused porous surfaces (SLIPS) concept was introduced in 2011 (Lafuma and Quéré 2011; Wong et al. 2011). Inspired by the *Nepenthes* pitcher, the slippery surface concept is based on infusing a low energy porous substrate with a lubricating liquid that has a strong chemical affinity and similar interfacial energies to the underlying substrate, wetting it completely and creating a stable, inert, and smooth lubricant overlayer on the surface (Figure 1e) (Preston et al. 2017, Tesler et al. 2015; Villegas et al. 2019; Yao et al. 2022). This novel technology can effectively repel aqueous and organic liquids, microorganisms, insects, and ice, making it suitable for a wide range of applications (Figure 1f,g). The premise of slippery coatings is that a liquid surface is inherently smooth and defect‐free down to the molecular level, thus reducing drag and the adhesion strength of contaminants (Applebee and Howell 2024). It is similar in many ways to the slimy, mucus‐coated scales of fish, which optimize drag and buoyancy and resist the accumulation of contaminants, resulting in reduced energy requirements for effective swimming. SLIPS function under high‐pressure conditions, self‐heal imperfections, provide optical transparency, reduce ice nucleation, ultra‐repellent to complex fluids such as crude oil and brine, and even repel highly contaminating biological media such as blood or biofilms during brief exposure (Amini et al. 2017; Cui et al. 2015; Epstein et al. 2012; Hou et al. 2015; Hou et al. 2018; Kim et al. 2013; Kovalenko et al. 2017; Leslie et al. 2014, Tesler, Kim 2015; Tesler et al. 2020, Villegas et al. 2019; Xiong et al. 2024).

However, SLIPS technology also has several substantial drawbacks. For instance, the infused lubricant that provides the slippery effect may deplete over time due to evaporation, shear‐induced and gravity drainage, and cloaking effect (Gunjan et al. 2021). This is a major challenge, impacting the long‐term performance of SLIPS systems in terms of stability and environmental protection. The most straightforward solution to this issue would be to adopt a set of lubricant parameters. Although various lubricants may be suitable for specific environments, they may still conflict with the desired outcome of the coating. For example, more viscous lubricants can be used to mitigate drainage in marine environments. However, this may result in a reduction in the anti‐biofouling efficacy of the coating (Kolle et al. 2022). Additionally, creating a porous/rough liquid‐infused surface, which is mechanically weaker than its bulk counterpart, typically requires multiple preparation steps. This, in turn, facilitates the development of novel strategies for substrate‐independent, easy‐to‐scale‐up, low‐cost, and adjustable SLIPS coatings (Deng et al. 2020). In response to the latter challenge, we have developed a novel, nondisruptive approach to form lubricant‐infused slippery surfaces (LISS) on bare materials using a “one‐pot” process (Tesler et al. 2021). This technology utilizes UV light of a specific wavelength to dissociate lubricant molecules that react with the surface, while the remaining molecules are used as the infusing lubricant. We have applied this technology to various metals, metal oxides, and ceramic materials. Plain polydimethylsiloxane (PDMS) oil has been used as an infused lubricant and low‐energy surface modifier. PDMS is readily available, inexpensive, and biocompatible (Barca et al. 2014). Our coatings showed good adhesion to all substrates with stability for more than a year underwater (Prado et al. 2023). The process is highly customizable, and the formed coating can be tailored to specific applications, particularly as an FR coating to prevent marine biofouling. In this context, we and others have shown that LISS/SLIPS coatings can equally prevent the adhesion of early marine colonizers, such as freshwater green algae and seawater diatoms, in laboratory short‐term exposure (Basu et al. 2020, Prado et al. 2023; Tesler et al. 2022; Xiao et al. 2013) but also in long‐term field studies (Kolle et al. 2022; Tong et al. 2024; Walter et al. 2024; Ware et al. 2018). Due to a rapid self‐healing process, LISS coatings have demonstrated superior surface protection even in the case of severe damage (Tesler et al. 2022). This innovation facilitates the protection of metallic surfaces not only from biofouling but also from corrosion and biocorrosion perspectives.

In summary, early anti‐biofouling coatings utilized toxic compounds, which were environmentally harmful. This led to the partial ban of certain biocidal substances and the increased regulation of others, resulting in the rapid development of more environmentally friendly approaches. Contemporary anti‐biofouling strategies are designed to minimize ecological impact while maximizing the efficacy of biofouling resistance. This includes nontoxic fouling release coatings inspired by natural mechanisms to deter organism attachment and facilitate their removal simply by changing surface properties of materials. In this regard, innovative approaches, such as aerophilic and liquid‐infused slippery surfaces, signify substantial advancements in addressing biofouling by acquiring distinctive surface properties aimed at impeding biofilm formation and/or facilitating facile fouling release. Despite the notable similarity between these technologies, there are also substantial differences. For instance, creating aerophilic surfaces requires two intrinsic components, which are high surface roughness and low‐energy coating. In exceptional cases of synergy between the two components, there is the potential for achieving thermodynamic stability of plastron, gaining surfaces sustainable performance underwater. However, the intrinsic weakness of aerophilic surfaces is high surface roughness, which reduces their mechanical resistance to abrasion. While the concept of slippery liquid‐infused porous surfaces (SLIPS) is predicated on analogous constraints of roughness and low‐energy coating, the air plastron is substituted by a lubricant, making these surfaces more sustainable to changes in environmental conditions. We and others have proved that liquid‐infused slippery surfaces can be constructed on bare smooth surfaces exhibiting good mechanical stability and comparable to SLIPS anti‐biofouling performance. These technologies have pros and cons, but they offer vital, non‐toxic alternatives for protecting surfaces from species attachment. They are adaptable for various applications, including in the marine and medical sectors. Despite recent advancements, challenges remain in ensuring the mechanical durability and long‐term effectiveness of both approaches. Our brief communication highlights the significant potential of these technologies, but continued research and development are essential for their refinement and broader implementation, as well as for enhancing their protective performance in diverse environments.

## Data Availability

Data are available on request from the authors.
